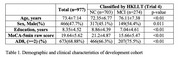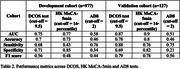# Development and validation of the DCOS (Digitalised Combined Objective‐Subjective cognitive screening): A five‐minute neuropsychological test for mild neurocognitive disorder

**DOI:** 10.1002/alz70860_100149

**Published:** 2025-12-23

**Authors:** Huijing Zheng, Jeanine Cheng, Ho Ko, Carol Y Cheung, Helen M. Meng, Adrian Wong, Vincent C.T. Mok, Bonnie Y.K. Lam

**Affiliations:** ^1^ Division of Neurology, Department of Medicine and Therapeutics, The Chinese University of Hong Kong, Hong Kong SAR, Hong Kong; ^2^ Lau Tat‐Chuen Research Centre of Brain Degenerative Diseases in Chinese, Faculty of Medicine, The Chinese University of Hong Kong, Hong Kong SAR, Hong Kong; ^3^ Li Ka Shing Institute of Health Sciences, Faculty of Medicine, The Chinese University of Hong Kong, Hong Kong SAR, Hong Kong; ^4^ Gerald Choa Neuroscience Institute, The Chinese University of Hong Kong, Hong Kong SAR, Hong Kong; ^5^ Margaret K. L. Cheung Research Centre for Management of Parkinsonism, Faculty of Medicine, The Chinese University of Hong Kong, Hong Kong SAR, Hong Kong; ^6^ Lam Kin Chung. Jet King‐Shing Ho Glaucoma Treatment and Research Centre, The Chinese University of Hong Kong, Hong Kong SAR, Hong Kong; ^7^ Department of Ophthalmology and Visual Sciences, The Chinese University of Hong Kong, Hong Kong SAR, Hong Kong; ^8^ Department of Systems Engineering and Engineering Management, The Chinese University of Hong Kong, Hong Kong SAR, Hong Kong; ^9^ Stanley Ho Big Data Decision Analytics Research Centre, Hong Kong SAR, Hong Kong

## Abstract

**Background:**

Early detection of mild neurocognitive disorder (NCD) enables timely and targeted interventions, especially before progression to major neurocognitive disorder. This study aims to develop a sensitive and simple digitalised screening tool, using artificial intelligence to capture and interpret spoken language, for the detection of mild NCD.

**Method:**

The Digitalised Combined Objective‐Subjective (DCOS) cognitive screening tool was developed based on DSM‐5 criteria, which includes subjective and objective cognitive impairment for the diagnosis of mild NCD. We developed and validated on two independent cohorts: a dementia speech biomarkers cohort (*n* = 977) for development and the Screening for Early Alzheimer's Disease Study cohort (*n* = 127) for validation. Mild NCD is defined using the Hong Kong List Learning Test (HKLLT with below 1SD cutoff; see Table 1). DCOS items were selected through ROC analysis from existing validated cognitive assessments, incorporating both subjective and objective domains, with the criteria that the items must be assessed using spoken language for the development of the digitalised tool. The scoring weights were calculated according to the estimated coefficients of a multivariate logistic regression model.

**Result:**

The new tool DCOS comprises six items (three subjective, three objective) with a maximum score of 16 points. In the development cohort, DCOS demonstrated good diagnostic accuracy (AUC=0.75) comparable to Hong Kong Montreal Cognitive Assessment‐5min (MoCA‐5min) (AUC=0.77) and superior to Ascertain Dementia 8 (AD8) (AUC=0.58). At the optimal cutoff score of 9.5, DCOS achieved a better balanced performance (F1=0.56) than both HK MoCA‐5min (F1=0.48) and AD8 (F1=0.44). External validation confirmed robust performance with improved diagnostic accuracy (AUC=0.87), achieving 88% sensitivity and 69% specificity (see Table 2). The sampling of the items and and the nature of the test has shown the feasibility of developing into a digitalised test.

**Conclusion:**

DCOS is a novel screening tool that assesses cognitive impairment from both subjective and objective domains. It presents a potential discriminative performance for mild NCD detection. The brief administration time (<5 minutes) makes it particularly suitable for primary health care. As a next step, DCOS will be incorporated with artificial intelligence and be applied on a digital platform to enhance its utility in large‐scale settings.